# Cases of Extirpation of the Ovaries

**Published:** 1830-04-01

**Authors:** 


### CASES OF EXTIRPATION OF THE OVARIES.

"Chirurgus medico quo differt? Scilicet isto,

"Enecat hic succis --enecat ille manu." *Max. Urentius.*

We are not among those who are ardent votaries of gastrotomy, nor do we consider the extirpation of an ovary or uterus as a mere bagatelle. At the same time, when cases of this description are recorded, it becomes our duty not to pass them in total silence.

*Case* 1.* A Polish woman, 40 years of age, of middling height, well formed, and of good constitution, applied to Dr. Dieffenback, of Berlin, on account of a tumour in the lower part of the abdomen. On examination the hypogastrium was found to be distended by a round tumour, moveable in every direction, and the abdominal parietes over its central part appeared to be only a quarter of an inch in thickness. The patient had been married since the age of 18, had always menstruated regularly, but never borne a child. The tumour had existed for ten or twelve years, and was attributed by the patient to the infliction of a blow and domestic annoyances. She was anxious to be rid of it, and applied to Dr. Dieffenback for that express purpose although several other surgeons had entirely discountenanced an operation. A consultation was called, and as is usual on such occasions, there were nearly as many different opinions as consultants, but the result of it was that Dr. Dieffenback performed the operation. An incision was commenced three inches above the umbilicus, carried down along the linea alba turning away from the navel to the left, and continued to within four or five inches of the symphysis pubis. The tumour was found to be in the interior of the peritoneum, and the latter was accordingly laid open at the superior part of the wound. A round, blueish-coloured tumour, of a cartilaginous consistence now occupied the opening in the peritoneum which was about four inches long. "*Unfortunately* an attentive examination proved that the tumour had a large base, containing vessels which pulsated strongly, and appeared to be attached to the spinal column." An opening which was made into the tumour gave rise to hæmorrhage which was only restrained by compression. The operator now found that he had caught a Tartar, and precipitately closed the wound in the belly which he had rashly, we had almost said ignorantly, made. Bad symptoms as vomiting, hiccup, &c. supervened, and it was only by active depletion and her lucky stars that the patient escaped from a fatal termination of her self-willed *divertissement*.

The foregoing case speaks so intelligibly for itself that remarks are almost unnecessary. We shall only say that no well-informed surgeon in this country would dare to cut into a woman's belly, under such circumstances as those detailed. The consequences of formidable operations so wantonly and foolishly engaged in, must be deserved disgrace to the surgeon, and worse than

---

* Rust's Magazin, B. 25, H. 2.

that, most probably destruction to the patient.

*Case* 2.* A female peasant was brought to bed with her eighth child at the age of 41, and two years afterwards her menses ceased to appear. From this time she experienced a dragging in the left hypochondrium, and a dull pain in the hypogastrium, attended with tumefaction of that region. At the age of 46 the abdomen was as much distended as at the ninth month of pregnancy, and M. M. Hopfer and Chrysmer who saw the patient, pronounced the disease to be disease of the left ovarium complicated with ascites. At the request of the unfortunate woman M. Chrysmer performed the operation. An incision of the integuments was commenced at the xyphoid cartilage and carried down to the symphysis pubis, turning to the left of the navel. The peritoneum was laid open for the same extent, and about eight pints of serous fluid evacuated, when the intestines and omentum protruded through the wound. The tumour was adherent to the colon, peritoneum, and stomach, and it took more than twenty minutes before these adhesions could be divided. The pedicle of the tumour which rested on the os ilii and arose from the broad ligament, was first tied with a double ligature, and then cut across between them. In thirty six hours after the operation the patient died of mortification of the intestines. On examining the extirpated ovary, it was found to be irregular and lobulated, weighed seven pounds and a third, was cartilaginous in some parts, and in others presented excavations filled with a stinking and greenish sanies, and finally in other parts still its texture was lardaceous. The right ovary was sound.

This is just the history of genuine ovarian disease, and just the results which would probably follow an operation in ninety-nine cases out of the hundred. As the ovarian tumour grows it almost always contracts adhesions with the neighbouring viscera and parts—adhesions and connexions, the separation and dissection of which is often extremely difficult in the dead body, and must be dreadful work in the living.

*Case* 3. A woman, æt. 38, had been brought to bed five times in the course of seven years. Subsequently to the fourth accouchement she suffered from inflammation of the uterus which lasted for several weeks, and from this time the patient complained of a dull pain in the left hypochondrium. A year and a half after her last accouchement a little tumefaction shewed itself in this region, disappeared for a while under the use of some sulphur baths, but returned and extended over the hypogastrium. In the course of two years her menstruation ceased, and was replaced by a foul and white discharge; which weakened the patient very much. M. Chrysmer was consulted, and recommended the operation which was performed as in the preceding case. Great part of the intestines escaped when the opening was made in the peritoneum, and were immediately enveloped in a warm and moist towel. The adhesions of the tumour to the peritoneum and brim of the pelvis were divided, its pedicle-like attachment to the broad ligament tied with two ligatures and cut across between them—the tumour then removed — the intestines transferred from the towel to their natural situation—the pelvis *sponged* out—and the wound united by the suture. The operation lasted a quarter of an hour, and immediately after its conclusion a nitrous emulsion was prescribed. A slight rigor and hiccup succeeded, but were calmed by a few doses of Sydenham's laudanum. Strange to say the patient survived, was able to return to her home in six weeks, and has since borne a healthy child. The tumour weighed eight pounds, was larger than the head of a child, was irregular, of livid colour in some places, and contained numerous cavities filled in part with a melliform matter, in part with a greenish and sanious fluid.

*Case* 4. An unmarried woman, 58

* This and the two following cases are from *Graefe and Walthers' Journal*, 12 B. 1 H.

years of age, small, affected with rickets since childhood, and who had suffered from chlorosis, fluor albus, irregular menstruation, and chronic affection of the liver, prayed M. Chrysmer to remove a large irregular tumour from the left iliac region, which made the abdomen look like that of a woman advanced in pregnancy. Besides this tumour the left lobe of the liver was felt enlarged, and an accumulation of serum was detected in the belly. M. Chrysmer, who appears to be quite the Coryphœus of ovariotomists, performed the operation as before. On opening the peritoneum nearly five pints of offensive serum, of greenish yellow colour, flowed from the wound. The tumour presented no adhesions except towards the sacro-iliac synchondrosis. The pedicle was four inches thick; it was tied and cut. Fainting fits succeeded and the patient expired in thirty six hours. On examining the body the peritoneum and intestines were found to be gangrenous, the uterus cartilaginous, the right ovary double its natural size, and tubercles in the right lobe of the liver. The tumour that had been extirpated weighed six pounds and a half, and its texture was lardaceous.

*Case* 5.* A girl, ætatis 24, was brought to bed in November 1824. In January 1825, the catamenia re-appeared and continued to flow regularly from this time till August, although they were more copious than natural, and accompanied with pain. In October she was thought to be pregnant again, but examination proved that she was not so, the belly being as large as in the eighth month. From the month of February the patient had profuse fluor albus and pains at first in the left iliac region, subsequently in the right. These symptoms had first appeared after connexion. The disease was considered to be encysted dropsy of the right ovary, and the tumour becoming more tense and fluctuating a puncture was made on the 15th December, and eight pints of serum evacuated. The pains ceased and the tumour diminished to the size of a fist, but on the 8th January the puncture was repeated; calomel, digitalis and conium administered; and frictions made with mercurial and digitalis ointment, as well as with that of the hydriodate of potass. These measures, however, failed of success, and on the 22d January the operation of paracentesis was again had recourse to, and the cyst afterwards injected with warm liquid consisting of one part of alcohol and eight of water. The injection was allowed to remain in the cyst for half an hour, the wound healed, and no bad consequences would seem to have ensued. Towards the middle of April, however, the belly again swelled and the tumour was harder than ever. Two punctures were made, but instead of serum portions of *omentum* came out and were removed by ligature. M. Martini, under whose care the patient was, now determined to operate, *because* he feared that his injection had occasioned a polypous or steatomatous degeneration of the tumour. This was what is vulgarly called "getting out of the frying pain into the fire," and repairing one faux pas by a worse.

However, an incision nine inches in length was made; the belly opened in the hypogastrium; and a smooth, round, white tumour, the size of a man's head, and apparently firmly fixed to the brim of the pelvis, was exposed. No pedicle was to be had, and a kind of cyst appearing at the superior part a trocar was plunged into it, and a pint of serous fluid discharged. The walls of the tumour collapsed, and some convolutions of intestine and a part of the omentum protruded. The operator not being able to separate the tumour from the bladder, the rectum and the pelvis, "was contented" (he could not well be otherwise) with removing the sac to prevent any fresh accumulation of fluid. Some arteries were tied and the wound brought together by suture. The first day passed off pretty well; on the second a considerable quantity of serum escaped by a canula which had been left at the inferior part of the wound; on the third day the discharge became bloody, prostration and hiccup, &c. set

* Rust's Magazin, B. 27, H. 3.

in, and the patient died "thirty-six hours" after the operation. On examining the body, the tumour was found to have extended into the hypogastrium, to be lardaceous in texture, and to contain "caverns filled with sanies." Below the tumour was a collection of bloody fluid. The disease was found to be situated in the left ovary, which by its morbid growth had extended to the right side and depressed the uterus. The fallopian tube and part of the broad ligament formed the peduncle of the tumour. No trace of inflammation was discovered in the abdomen, so, says the narrator of the case, it is probable that the patient died from the quantity of the bloody and serous discharge. It is much more probable that she died from the shock and irritation of so formidable and ill-judged an operation.

Our readers can form their own opinions on the foregoing cases: we certainly have formed our's. One only out of five was cured, and a miracle that seems to have been; and in two out of the remaining four, the operation was abandoned when half done, in consequence of the difficulties and obstacles that presented themselves. To those much acquainted with morbid anatomy this will not appear at all surprising; on the contrary, the wonder with them will be, that the operators managed as well as they did. No man can certainly tell before he opens his patient's belly, whether the tumour has a stalk or whether it has not; whether it be extensively adherent to the pelvic and abdominal viscera or otherwise; whether, in short, he will be able to complete an operation the propriety of which is questionable at the best, or whether he may be compelled to arrest his hands in the middle of it. This is a fearful prospect to a conscientious surgeon, for it is almost too late when the interior of the abdomen is displayed to pronounce that the case is not one for the knife. But there is yet another circumstance to "give us pause." Are we always sure of the nature of an abdominal tumour—have ovarian diseases never been mistaken? A very eminent operator performed gastrotomy for ovarian tumour, but found none; an uterus was lately removed in Paris, and discovered to be healthy! Will such facts have no weight on the minds of the profession? Do not the rash remedies of the physician, and the desperate attempts of the surgeon almost authorize the sarcastic observation of Urentius quoted as the motto to this article, and which concludes thus:—

"Carnifice hoc ambo, tantum differre videntur:—
"Tardius hi (medici) faciunt, quod facit ille (chirurgus) cito."

